# Diffusion of Messages from an Electronic Cigarette Brand to Potential Users through Twitter

**DOI:** 10.1371/journal.pone.0145387

**Published:** 2015-12-18

**Authors:** Kar-Hai Chu, Jennifer B. Unger, Jon-Patrick Allem, Monica Pattarroyo, Daniel Soto, Tess Boley Cruz, Haodong Yang, Ling Jiang, Christopher C. Yang

**Affiliations:** 1 Department of Preventive Medicine, University of Southern California, Los Angeles, California, United States of America; 2 College of Computing and Informatics, Drexel University, Philadelphia, Pennsylvania, United States of America; University of Waterloo, CANADA

## Abstract

**Objective:**

This study explores the presence and actions of an electronic cigarette (e-cigarette) brand, Blu, on Twitter to observe how marketing messages are sent and diffused through the retweet (i.e., message forwarding) functionality. Retweet networks enable messages to reach additional Twitter users beyond the sender’s local network. We follow messages from their origin through multiple retweets to identify which messages have more reach, and the different users who are exposed.

**Methods:**

We collected three months of publicly available data from Twitter. A combination of techniques in social network analysis and content analysis were applied to determine the various networks of users who are exposed to e-cigarette messages and how the retweet network can affect which messages spread.

**Results:**

The Blu retweet network expanded during the study period. Analysis of user profiles combined with network cluster analysis showed that messages of certain topics were only circulated within a community of e-cigarette supporters, while other topics spread further, reaching more general Twitter users who may not support or use e-cigarettes.

**Conclusions:**

Retweet networks can serve as proxy filters for marketing messages, as Twitter users decide which messages they will continue to diffuse among their followers. As certain e-cigarette messages extend beyond their point of origin, the audience being exposed expands beyond the e-cigarette community. Potential implications for health education campaigns include utilizing Twitter and targeting important gatekeepers or hubs that would maximize message diffusion.

## Introduction

Use of electronic cigarettes (e-cigarettes) has increased rapidly in the past decade [1]. E-cigarettes are nicotine delivery devices that heat a liquid to create an aerosol, which can then be inhaled [2]. E-cigarette sales in the United States more than doubled between 2012 and 2013, from $273 million USD to $636 million, and are expected to continue growing [3]. Tobacco companies are capitalizing on this new product by introducing their own e-cigarette brands (e.g. Reynolds launching VUSE) or acquiring smaller e-cigarette companies (e.g. Lorillard purchasing Blu).

Consensus has not been reached over e-cigarettes’ long term public health impact [4]. As research on the potential risks [5,6] and benefits [7,8] of e-cigarettes continues to accumulate, it is important to understand how the public obtains information that may influence decisions about whether to use these products. Information about new products is increasingly disseminated through social media such as Twitter, YouTube and Facebook [9]. Twitter is a popular micro-blogging platform, affording users the ability to broadcast short messages (tweets) to large audiences. Twitter users can view these tweets and pass them along (retweet) to their own followers. Retweeting messages in networks of “friends of friends” makes it possible for any tweet to reach more people than the original author’s immediate network and to cross over to new networks. Earlier studies have demonstrated the diffusion process in retweet networks and the ability for certain messages to go viral [10,11].

The ability of Twitter users to quickly and easily forward messages to their followers affords tweets about e-cigarettes further reach than the original marketing audience. This becomes a proxy for selective filtering; the first group of users who read the full selection of all e-cigarette messages can choose to retweet only those that they deem important or relevant. Advertising in social media takes advantage of properties such as retweeting, although few studies have examined e-cigarette marketing specifically. Huang et al [12] captured tweets related to e-cigarettes and analyzed their content and user profiles. They classified 90% of the tweets as messages from commercial based sources, concluding that Twitter served as an important platform for e-cigarette marketing. Chu et al., [13] found that flavor-related tweets by e-cigarette companies were more frequently retweeted compared to messages without flavor mentions. The present study was informed by both reports and aimed to understand the audience that these commercial messages reached as well as the retweet potential of each message.

This study examined how messages from an e-cigarette brand reached different types of people, including e-cigarette users, retailers, and advocates as well as people without discernable opinions about e-cigarettes. We identified the social network of Twitter users who retweeted messages from an e-cigarette brand (Blu) and the followers of those users who retweeted the messages again to their own social networks. Using this social network, we investigated the following research questions:

Research Question 1 (RQ1): How does the size of the social network change over time?
Research Question 2 (RQ2): How do the types of Twitter users (e.g., e-cigarette users or advocates, individuals who have not necessarily taken a position on e-cigarettes, or businesses) that are reached change as the network expands?
Research Question 3 (RQ3): How do the prevalent messages change as followers receive messages from the e-cigarette brand and selectively retweet certain messages to their own followers?

## Methods

All data collected were publicly available (see data in S1 Table). No personally identifiable information is included in this study. We collected three months of Twitter data (February 1, 2014 to April 30, 2014) based on tweets and retweets from the Blu e-cigarette brand (owned by Lorillard, then by Reynolds Tobacco and finally Imperial Tobacco in 2014). User data were retrieved at the end of April. We chose Blu because it was the brand leader in the e-cigarette market in 2014, had a high level of Twitter activity, and the highest advertising expenditures for e-cigarettes, comprising over 75% of all e-cigarette advertising in 2012 [14]. We utilized the Twitter REST API v1.1 to collect data from Twitter and constructed the @blucigs retweet network. Retweets were identified via the API’s *statuses/retweets* method.

**Fig 1:**
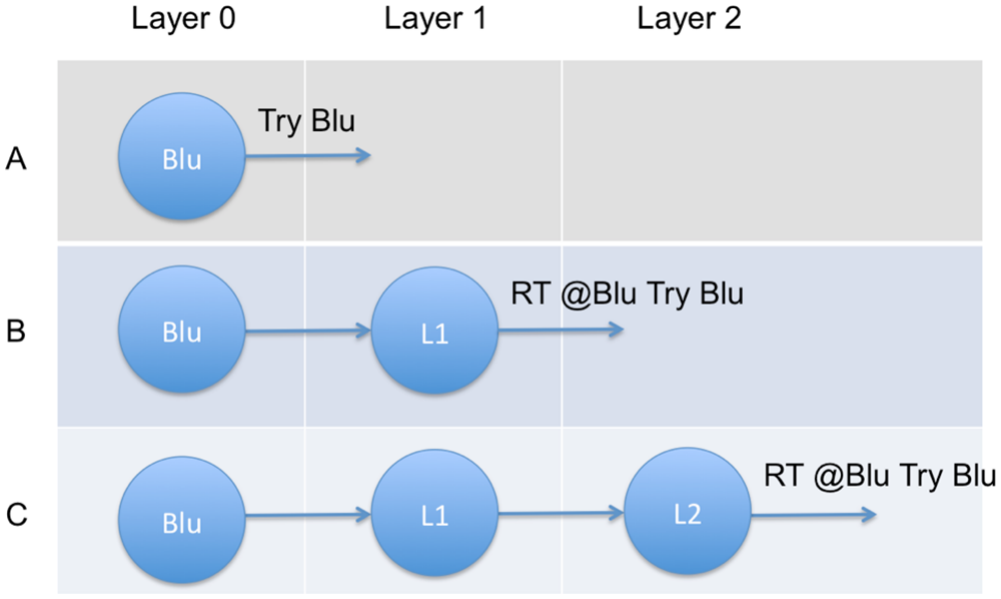
Description of the 3-layer retweet network. (A) Layer 0 (Blu) sends the original tweet. (B) This is followed by a Layer 1 user that retweets the message. (C) Finally, a Layer 2 user retweets the retweet.

The Twitter network was generated with three layers of users. Each layer constitutes the distance a tweet has traveled via the retweet mechanism. We followed tweets originating from Blu (Layer 0) and retweeted by Blu’s followers (Layer 1). The tweets selected by Layer 1 users to be retweeted were seen by their respective followers (Layer 2), who could choose to retweet the messages to their own followers. To address RQ1 (i.e., change in network size), at each layer we captured the content of the messages and meta-information of the senders. This type of analysis resulted in construction of a 3-layer ego-centered retweet network, where Layer 0 (the ego) is Blu, Layer 1 is the retweeters of Blu’s messages, and Layer 2 is the retweeters of Layer 1’s retweets of Blu’s messages (Fig 1).

We applied metrics from social network analysis to help identify features in the structural organization of the network [15]. We focus specifically on modularity, which describes how well a network can be divided into smaller clusters [16]. Modularity is useful in finding community structures by determining if a person has more internal ties to a local cluster than external ties to other clusters of Twitter users [17]. A visualization tool (Gephi) generated the retweet network. The network graph visually represents users as small circles, or *nodes*, and retweet actions as lines connecting them, or *edges*.

Next, we developed a coding scheme for the user profiles. We first extracted the profile description information of each Twitter user in the study. Afterwards, two of the authors in the study (KC and TC) conducted an initial discussion and exploration of 200 random profiles (out of approximately 3400) to create a coding scheme through an inductive process. This resulted in a decision to classify users into one of the following categories:

- Person-Supporter: profiles of persons who mention e-cigarettes or other related terms (e.g. vaping) in a supportive manner
- Person-BasicProfile: profiles of persons who do not mention e-cigarettes or other related terms
- Researcher: profiles of persons who mention that they conduct research on tobacco
- Nonperson: profiles of entities that were not individuals and had no affiliations with e-cigarette brands or companies (e.g. musical band)
- Industry-Retailer/Manufacturer: profiles of e-cigarette retailers or manufacturers
- Industry-Other: profiles of e-cigarette affiliated company (e.g. vaping magazine, online marketer, etc.)
- Unclassified: profiles were blank or contained only symbols

After the categories were established, two authors (KC and MP) conducted a profile analysis on the full list of users. In addition to the user profile data, we also extracted meta-information about each user, including the number of other users they are following, the number of followers they have, number of tweets posted, and when they joined Twitter.

Analyses of individual tweets could have been used for categorization instead of profile descriptions. However, we believe that users who self-identify (i.e. describe in their profile) as an advocate, or user, of e-cigarettes is the most reliable measure to use in this study. While Twitter users might tweet or retweet about many topics, it is difficult to gauge–or more importantly, compare–their sentiment toward any particular topic based on a varying number of posts. Our method of categorization likely has high sensitivity but low specificity where those who explicitly state they use e-cigarettes in their profile are certainly users and those who do not state in their profile that they use e-cigarettes may still use or advocate for e-cigarettes. Notwithstanding this bias, we believe this categorization strategy best distinguishes between users with different levels of advocacy, or use, and provides a clear boundary to study the diffusion of messages through distinct user categories.

To address RQ2 (i.e., regarding the types of Twitter users being reached), we conducted a chi-square test to determine whether the proportion of retweeters in each profile category differed between Layer 1 and Layer 2. We also combined the network modularity with the profile classifications to examine whether clusters found in the retweet network were associated with the types of users.

To answer RQ3 (i.e., how messages change), we examined the content of the tweets transmitted by each layer of users, and compared word frequencies to determine if there were changes in topics.

## Results

Table 1 presents the number of users in each layer and the number of links in each retweet network generated from the data collected in February, March, and April of 2014, as well as the February-April period overall. It shows that the number of Layer 1 users who retweeted messages from @blucigs increased from 40 in February to 100 in April, representing an increase in retweet activity. Similarly, the number of Layer 2 users who retweeted the messages that they received from Layer 1 users increased from 381 in February to 1082 in April. The total number of messages tweeted by these Twitter users increased from 687 in February to 1304 in April. Examples of collected tweets included:

(Layer 0) “@USERNAME Hope you're enjoying #SXSW! We'd like to give you VIP access to our Electric Lounge. Follow us so we can DM you the Link/ PWD!”
(Note: SXSW is the acronym for the South by Southwest music festival, where Blu had a marketing presence, including a Freedom Lounge where people could sample Blu products.)
(Layer 0) @USERNAME Awesome! Hope to see you at the freedom lounge, too!
(Layer 1) “RT @USERNAME: Unfortunately this is how many of our leaders choose to look at #ecigs http://t.co/58bE8mlN7H”
(Layer 1) “RT @USERNAME: #WA bill to tax #ecigs 95% does not pass! #goodnews #vaping #vapelife #vapenews”
(Layer 2) “RT @USERNAME: BBC: Test finds #ecigs contain no discernible toxins or carbon monoxide http://t.co/qcrUg8hu84”
(Layer 2) “RT @USERNAME: Have You Read This Yet? Unlucky Strike—What Big Tobacco May Mean For Vaping http://t.co/HNY2cbQlc0”

**Table 1:** General network information.

Period	Feb 1–28	Mar 1–31	Apr 1–30	Feb 1 –Apr 30
# of nodes Layer 0	1	1	1	1
# of nodes Layer 1	40	93	100	214
# of nodes Layer 2	381	609	1082	1956
Total nodes	422	703	1183	2171
Total edges (retweets of messages)	687	762	1304	2684

The number of nodes (i.e. users) in Layer 0, Layer 1, and Layer 2, the total number of nodes and edges (i.e., retweets) in the retweet network constructed in February, March, and April of 2014. The increase for each layer over each month represents an increase in retweet activity.

Table 2 presents the descriptive statistics of the users in Layer 1 of the retweet network constructed from February to April of 2014. The variation in the number of followers covers a large range, from 0 to 116,033. Additionally, each Layer 1 user brings a median of 187 new followers who can be exposed to retweeted e-cigarette messages.

**Table 2:** Descriptive statistics of the users in Layer 1 in the retweet network.

	Followed	Followers	Tweets
Mean	509	1809	7724
Median	254	187	1530
Mode	1	3	26
Std. Deviation	600	9820	17605
Range	3197	116033	107272
Minimum	0	0	0
Maximum	3197	116033	107272

**Fig 2:**
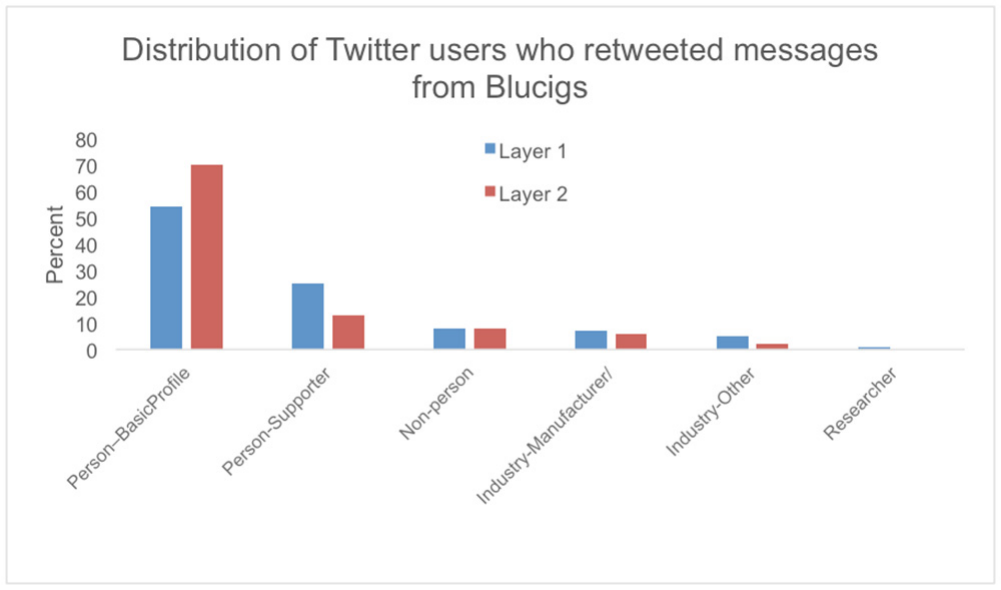
The number of users found in each category in Layer 1 and Layer 2 of the retweet network.

Fig 2 presents the number of users found in each category in Layer 1 and Layer 2 of the retweet network, i.e. users who retweeted messages from Blu, and users who again retweeted the retweets. In both layers, the most frequent type of user was Person-BasicProfile, individuals who did not specifically say in their profiles that they used or supported e-cigarettes. The proportion of users in this category was significantly larger in Layer 2 compared to Layer 1 (chisq _(5)_ = 215.71, p < 0.001).

**Fig 3:**
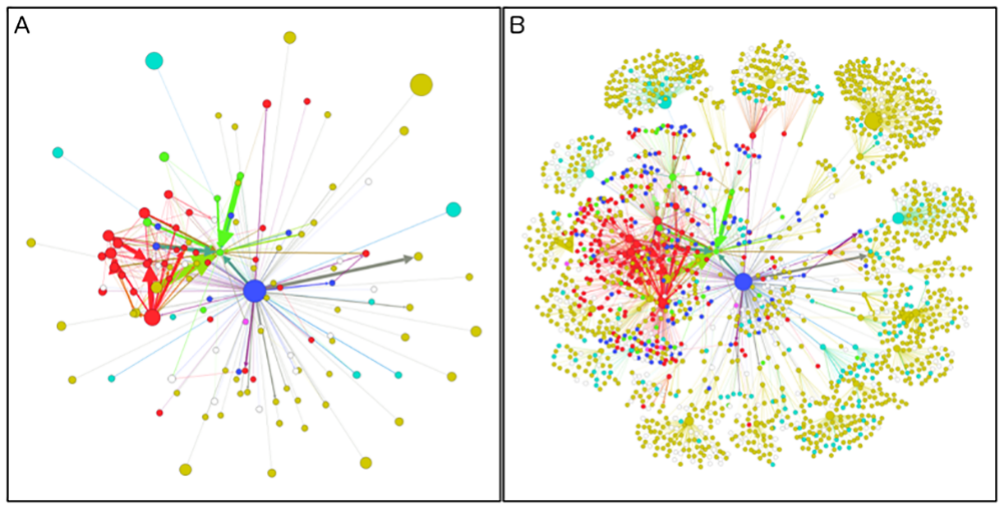
The retweet networks of the data collected February to April of 2014. In the rewteet network, the size of node corresponds to the number of retweets from this particular user and the width of link corresponds to the number of retweets made by the users of the ending node (*y*) from the users of the starting node (*x*). Red = Person-Supporter, Blue = Industry-RetailerManufacturer, Yellow = Person-BasicProfile, Cyan = Nonperson, Green = Industry-Other, White = Unknown, Purple = TobaccoControl-Research. (A) Includes users from Layer 1 (i.e., only those who retweeted messages by Blu) and (B) includes all users (i.e. Layer 1 and Layer 2).

Fig 3 shows the network graphs depicting two layers of the Blu retweet network. Fig 3A is the Layer 1 retweet network, containing only followers of Blu. Fig 3B is the retweet network showing both followers of Blu (Layer 1) and followers of Blu followers (Layer 2). The classification of users in two levels of Blu’s retweet network shows a striking difference in persons who explicitly mention support of e-cigarettes (colored red) and those who do not mention e-cigarettes. From Layer 1 to Layer 2, the proportion of Person-supporters dropped almost 50%, whereas the proportion of Person-BasicProfiles increased over 30%.

Table 3 shows the word frequencies of the top 10 terms at each layer of the Blu retweet network. Layer 0 describes the messages from Blu, and several of the top 10 terms are related to Blu-sponsored events, including *lounge*, *#sxsw*, and *vip*. The top term *dm* is an abbreviated form of “direct message”, and used by Blu as instructions for its followers on how to contact the brand’s representatives. Each of these, and other top frequency words, refer to Blu’s sponsored event presence at the South by Southwest music festival held March 7–16, 2014. Layer 1 describes the messages that were retweeted from Blu, and Layer 2 describes messages that were retweeted by followers of Blu’s followers. These terms are more focused on e-cigarettes in general (e.g. #*ecigs*, #*vaping*), as well as terms about e-cigarette news (e.g. *#vapenews*, *ban*).

**Table 3:** Top terms found by users in each layer, with raw frequencies.

Layer 0	Freq (N = 4810)	Layer 1	Freq (N = 2471)	Layer 2	Freq (N = 28743)
dm	175	#ecigs	121	#ecigs	626
lounge	125	#vaping	61	ban	191
follow	123	#vapenews	48	cigarettes	185
vip	119	Tobacco	34	new	157
#sxsw	116	#wa	32	smoking	148
electric	111	#ecig	31	#vaping	142
link	110	blu	30	#ecig	139
access	110	pass	30	cig	130
pwd	109	choose	29	cigarette	126
enjoying	63	#vapelife	26	quit	126

A network analysis computed the modularity of the graph to be 0.801, indicating a high degree of clustering in the network. There was a significant relationship between each node’s modular cluster and its user profile classification (chi2 _(258)_ = 1300, p < 0.001).

## Discussion

This study examined diffusion of messages from the Blu e-cigarette brand to its followers on Twitter. The number of people reached by Blu’s tweets expanded 10-fold from Layer 1 to Layer 2. It was unclear if Blu’s messages continued to expand at the same rate in subsequent layers, or slowed after Layer 2 as messages saturated among people who may be uninterested in e-cigarettes. In addressing RQ1 (i.e., change in network size over time), data also showed an increase in each layer every month. As with the change from Layer 1 to Layer 2, this was an exponential increase; as Blu gained more retweeters, each user brought their entire network of followers (median of 187 followers per user), presenting a new community for potential exposure to e-cigarette messages.

The variation in the number of Layer 1 followers covered a large range, from 0 to 116,033, suggesting that many different types of users, from casual to active tweeters, exist in Blu’s network. From Layer 1 to Layer 2, Blu’s messages diffused beyond its main supporters and to members of the general public who were not necessarily e-cigarette advocates or users. These findings helped address RQ2 about how the types of users reached by Blu changed as the network expanded. For example, Layer 1 profiles showed a ratio of Person-BasicProfiles to Person-supporters at nearly 2-to-1, while the change to Layer 2 brought the ratio to greater than 5-to-1. These Person-BasicProfiles were Twitter users who may not specifically seek out information on e-cigarettes, but were exposed to e-cigarette messages and advertising via their friends who retweet Blu’s messages. Data did not indicate the characteristics of Person-BasicProfiles; they could be smokers, e-cigarette users, male, female, old, or young. However, while the Person-supporters explicitly supported e-cigarettes in their profiles, the lack of explicit support in the Person-BasicProfile category means there is the possibility of exposing messages to non-vapers or youth, potentially increasing their interest in nicotine products [18].

The high modularity (0.801) revealed that there were very distinct sub-clusters in the retweet network. For comparison, a retweet network of political communication scored 0.48 [19] and a clustering study of different languages on Twitter resulted in a measure of 0.81 [20]. This has implications for both the Twitter network analysis and e-cigarette users. In the retweet network, a high modularity would suggest that the followers for most of the users have little overlap. As each message is retweeted by a user to their followers, it is likely these new recipients only retweet from one user even if they are exposed to the retweets from other users in the previous layer. These low-overlapping followers would result in more clustering in the network, which we displayed graphically in Fig 3.

Table 3‘s top terms showed how messages in Twitter change during the diffusion process (RQ3). In contrast to the tweets from Blu (Layer 0), which focused on social and entertainment events, Layer 1 users were more likely to retweet about e-cigarette news (e.g. *#vapenews*) and laws (e.g. *#wa*, related to e-cigarette legislation in the state of Washington). The Layer 2 top terms also included #*ecigs* and #*vaping*, as seen in Layer 1, although the change in usage–where *#ecigs* increased and *#vaping* decreased–showed that messages became more focused on ecigs. This presents an interesting distinction, as “ecigs” is the term for the product or technology, whereas vaping describes the action of using an e-cigarette. A shift in frequency suggested that Layer 2 users were more interested in the product rather than the activity. Alternatively, vaping is a term that is more likely to be used by individuals more familiar with electronic nicotine delivery systems of various kinds. This suggests that Layer 2 users may not identify with certain messages disseminated by Blu’s followers. Layer 2 users may be unfamiliar with terms commonly used by avid e-cigarettes users. The change in high frequency terms throughout the diffusion process demonstrates how users at different levels in a retweet network can serve as proxy filters. In other words, the focus of messages shifted from product advertisement (Layer 0) to social behavior (Layers 1 and 2).

The network analysis also revealed clustering by Person-supporters (colored red in Fig 3). While moderately visible at the first level of retweeters (Fig 3A), it becomes easy to distinguish when looking at the full network (Fig 3B). This suggested that many of Blu’s tweets are being retweeted within the sub-network of Person-supporters, and the inherent nature of the Twitter network might expose users to a disproportionate number of messages that reinforce their current beliefs or preferences. The idea that the Internet reinforces prior beliefs due to selective exposure has been well described in prior research [21,22]. When health education campaigns are created and disseminated via social networking sites, campaign designers should consider how they plan to reach individuals with pre-existing opposing beliefs before the campaign is implemented. Additionally, campaign designers could consider how the campaign reach is expanded by those who are first exposed, and in turn, spread information to others. Other studies [23] have also studied patterns of use on Twitter in connection to e-cigarette policies, and developed suggestions in how public health organizations can coordinate strategies.

The change in word frequencies indicated that many of Blu’s original messages, especially those related to the SXSW event, likely did not diffuse beyond their Person-supporter sub-network or beyond the event date. Rather, the more generic e-cigarette tweets would be the ones that spread to the Layer 2 users. While the Layer 1 users (mostly Person-supporters and both Industry types of users) were exposed to Blu’s direct messages about advertisements and events, it was the messages regarding news, policies, and generic e-cigarette content that were reaching the Person-BasicProfiles. The overall result of this selective retweet pattern is that Blu can tweet primarily upbeat, non-controversial messages about entertainment and social events, and their followers will filter this content and disseminate the generic policy messages.

### Limitations

Data were based on all tweets originating from @blucigs during a three-month period in 2014. As such, they may not represent the types of messages sent at another time period or from another commercial source. Relatedly, retweets of posts made during the study period, but occurring after April 2014, were not captured. Additionally, we based our user classification analysis on the user’s Twitter description, which can be any text submitted by the user. This introduced a bias toward the most vocal advocates. In other words, not all supporters of e-cigarette use explicitly state this fact in their profile description. However, we believe how the user self-identifies is the most important factor in this study, although it could be further supported by examination of their tweets. An expansion of this type of study could take advantage of new research in applying machine learning techniques to classify tweets [24], which would allow for a much larger dataset. We could not draw conclusions about the demographics of the audiences (e.g., percent youth exposed to marketing messages), or about the effects of these messages on behavior. Future studies could potentially take advantage of research from the natural language processing field that has developed methods to infer user attributes based on tweet content [25,26].

## Conclusions

Despite these limitations, findings provided insights about the spread of brand marketing messages through person-to-person messages on the Internet. The retweet network in our data demonstrated how rapidly and widely messages diffused, reaching an exponential number of users. By the second level of followers, there was a large change in the types of users who were seeing the messages, exposing those who might not explicitly support e-cigarettes, or potentially vulnerable populations such as youth. User filtering of retweets also showed how the content changes at each level of followers, going from sponsored events to politics, news, and vaping.

The diffusion of tobacco-related marketing messages through social media suggests a need for real-time surveillance of brand marketing messages through Internet channels that do not require age-verification. Pending consensus on the public health impacts from e-cigarettes, restrictions may need to be placed on how e-cigarette companies market their products via social media. Moving forward, research is needed to better understand the impact of marketing messages on social media users in order to better inform education campaigns.

## Supporting Information

S1 Table All tweet ID's by month and layer. (XLSX)
